# The endocannabinoid system in appetite regulation and treatment of obesity

**DOI:** 10.1002/prp2.70009

**Published:** 2024-09-18

**Authors:** Marija Kurtov, Igor Rubinić, Robert Likić

**Affiliations:** ^1^ Department of Clinical Pharmacology and Toxicology University Hospital Sveti Duh Zagreb Croatia; ^2^ Department of Clinical Pharmacology and Toxicology Clinical Hospital Centre Rijeka Rijeka Croatia; ^3^ University of Rijeka, School of Medicine Rijeka Croatia; ^4^ Department of Clinical Pharmacology and Toxicology Clinical Hospital Centre Zagreb Zagreb Croatia; ^5^ University of Zagreb, School of Medicine Zagreb Croatia

## Abstract

The endocannabinoid system (ECS) is a complex cell‐signaling system that is responsible for maintaining homeostasis by modulating various regulatory reactions in response to internal and environmental changes. The influence of ECS on appetite regulation has been a subject of much recent research, however, the full extent of its impact remains unknown. Current evidence links human obesity to ECS activation, increased endocannabinoid levels in both central and peripheral tissues, along with cannabinoid receptor type 1 (CBR1) up‐regulation. These findings imply the potential pharmacological use of the ECS in the treatment of obesity. Here, we present various pathophysiological processes in obesity involving the ECS, highlighting different pharmacological options for modulating endocannabinoid activity to treat obesity. However, the potential of those pharmacological possibilities remains under investigation and requires further research.

## Graphical abstract

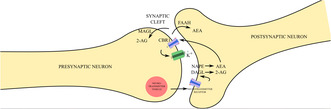

**Abbreviations**
2‐AG: 2‐arachidonoylglycerol
2‐AGE: 2‐arachidonyl glyceryl ether
AEA: N‐arachidonoylethanolamine
AgRP: Agouti‐related protein
AMPK: AMP‐activated protein kinase
BMI: body mass index
CBR: cannabinoid receptors
CBR1: cannabinoid receptor type 1
CNS: central nervous system
DAGL: diacylglycerol lipase
ECS: endocannabinoid system
FAAH: fatty acid amide hydrolase
FDA: Food and Drug Administration
MAGL: monoacylglycerol lipase
MAPK: mitogen‐activated protein kinase
mTOR: mammalian target of rapamycin
NADA: N‐arachidonoyl dopamine
NAGLy: N‐arachidonylglycine
NAMA: negative allosteric modulating antibody
NAMs: negative allosteric modulators
NAPE‐PLD: N‐acyl phosphatidylethanolamine‐specific phospholipase D
NASH: non‐alcoholic steatohepatitis
NPY: neuropeptide Y
ODA: cis‐9,10‐octadecanoamide
OEA: oleoylethanolamide
PEA: palmitoylethanolamide
POMC: pro‐opiomelanocortin
PPARs: peroxisome proliferator‐activated receptors
SEA: stearoylethanolamide
STAT3: signal transducer and activator of transcription 3
THC: delta 9‐tetrahydrocannabinol
TRP: transient receptor potential
UCP2: mitochondrial uncoupling protein 2
USA: United States of America
WHO: World Health Organization
α‐MSH: α‐melanocyte‐stimulating hormone

## 1 INTRODUCTION

The endocannabinoid system (ECS) is a complex biological network responsible for maintaining homeostasis by modulating various regulatory reactions in response to internal and environmental changes.^1^, ^2^, ^3^ This complex signaling system is present throughout the human body and includes endocannabinoids, cannabinoid receptors (G‐protein‐coupled receptors), and enzymes responsible for synthesizing and degrading endocannabinoids.^1^, ^2^, ^3^

The ECS is important in regulating different range of functions such as nociception, appetite regulation, memory and learning, immune response, fertility and reproduction, glucose and lipid metabolism thus making it an interesting target for treating many conditions, including obesity.^1^, ^2^, ^4^ The underlying molecular mechanisms of ECS dysregulation in obesity need clarification to better understand the relationship between ECS and obesity. With obesity reaching pandemic levels, there is an urgent need for new therapeutic options. Current evidence suggests that targeting the ECS might present a viable option.

## 2 THE ECS

Endocannabinoids are synthetised by postsynaptic neurons in response to excessive neurotransmitter release functioning as negative feedback regulators that inhibit further neurotransmitter release. The first endocannabinoids to be discovered, N‐arachidonoylethanolamine (AEA) and 2‐arachidonoylglycerol (2‐AG), are the most extensively studied.^5^ Both are synthesized on‐demand from the lipid membrane of the postsynaptic neuron via multiple pathways, with 2‐AG synthesis being crucially dependent on the enzyme diacylglycerol lipase (DAGL), while AEA synthesis involves multiple enzymes.^1^, ^2^, ^6^ An increase in the intracellular Ca^2+^ prompts their release into the synaptic cleft by passive diffusion, where they bind to cannabinoid receptors (CBR) present on the presynaptic neuron membrane. There are two types of cannabinoid receptors, CB1 and CB2, both of which are G‐protein‐coupled receptors that, upon activation, inhibit adenylyl cyclase and trigger mitogen‐activated protein kinase (MAPK) signaling.^7^ CB1 receptors are primarily located on presynaptic terminals, where they inhibit voltage‐dependent Ca^2+^ channels and activate K^+^ channels. In contrast, CB2 receptors are predominantly found on postsynaptic membranes and influence the same channels.^7^, ^8^ AEA acts as a partial agonist of CBR1 and has a lower affinity for CBR2, whereas 2‐AG, present in much higher concentrations in the brain, is a full agonist of both CBR1 and CBR2.^3^, ^9^, ^10^ CBR1 can be found in most human tissues, predominantly in the central nervous system (CNS), but also in the adipocytes, endocrine glands, gastrointestinal and other tissues, while CBR2 is primarily expressed in immune cells, but also present in neurons and glia within the CNS.^1^, ^2^, ^5^, ^11^, ^12^ Several other receptors, such as transient receptor potential (TRP), peroxisome proliferator‐activated receptors (PPARs), nicotinic, glycine, GABA, and G‐protein coupled receptors 18 and 55, among others, can also be engaged by the endocannabinoids, yet the impact is not yet completely clear.^13^

**FIGURE 1:**
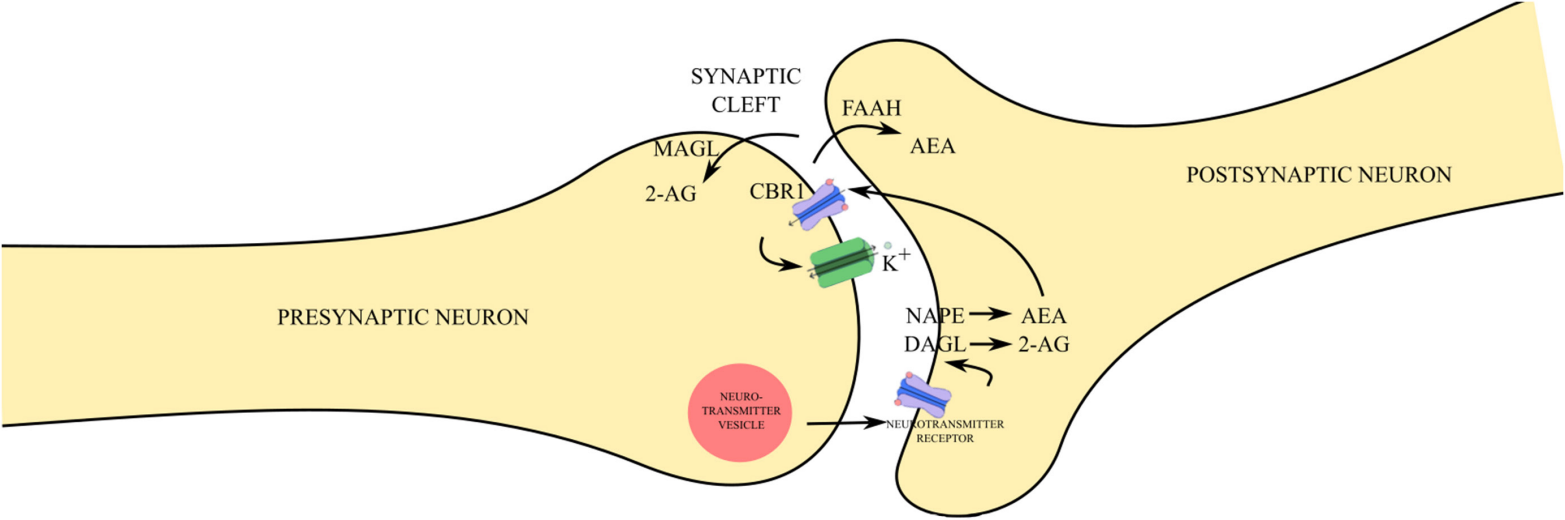
Simplified overview of ECS signaling. A neurotransmitter is released from a vesicle in the presynaptic neuron into the synaptic cleft due to Ca2+ influx, where it binds to its specific receptor on the membrane of the postsynaptic neuron. This binding activates the enzymes NAPE‐PLD and DAGL, which synthesize AEA and 2‐AG, respectively, in the postsynaptic neuron. Both AEA and 2‐AG are hydrophobic, allowing them to diffuse from the postsynaptic membrane. AEA and 2‐AG then travel back across the synaptic cleft and bind to the CBR1 receptor on the presynaptic neuron. The CBR1 receptor is linked to the Gi protein, which inhibits the voltage‐gated Ca2+ channel, causing membrane hyperpolarization by stimulating K+ channels and reducing depolarization. This process decreases Ca2+ influx, thereby decreasing neurotransmitter exocytosis. Subsequently, 2‐AG undergoes passive diffusion into the presynaptic membrane, where it is degraded by MAGL, while AEA undergoes passive diffusion into the postsynaptic membrane, where it is degraded by FAAH. 2‐AG ‐ 2‐arachidonoylglycerol; AEA ‐ N‐arachidonoylethanolamine; CBR1 – cannabinoid receptor type 1; DAGL ‐ diacylglycerol lipase; FAAH ‐ fatty acid amide hydrolase; MAGL ‐ monoacylglycerol lipase; NAPE‐PLD – N‐acyl phosphatidylethanolamine‐specific phospholipase D.

Upon the activation of CBR1, hyperpolarization of the presynaptic neuron occurs thus causing a reduction in neurotransmitter release. AEA and 2‐AG are rapidly hydrolysed by fatty acid amide hydrolase (FAAH) and monoacylglycerol lipase (MAGL), respectively, into inactive compounds.^1^, ^2^, ^14^ The process is illustrated in Figure 1.

Several other endocannabinoids, including 2‐arachidonyl glyceryl ether (2‐AGE), N‐arachidonoyl dopamine (NADA), oleamide (cis‐9,10‐octadecanoamide [ODA]), N‐arachidonylglycine (NAGLy), palmitoylethanolamide (PEA), stearoylethanolamide (SEA), and oleoylethanolamide (OEA) have been identified, although their roles remain less understood.^1^, ^2^, ^12^

K^+^ channels and reducing depolarization. This process decreases Ca^2+^ influx, thereby decreasing neurotransmitter exocytosis. Subsequently, 2‐AG undergoes passive diffusion into the presynaptic membrane, where it is degraded by MAGL, while AEA undergoes passive diffusion into the postsynaptic membrane, where it is degraded by FAAH.

## 3 THE ECS AND OBESITY

Obesity is a complex, multifactorial disease influenced by genetic, metabolic, social, behavioral, and cultural factors leading to chronic positive energy balance.^15^ The World Health Organization (WHO) defines obesity as abnormal or excessive fat accumulation that presents a health risk. The body mass index (BMI) is commonly used to assess obesity, with a BMI over 25 classified as overweight, and over 30 as obese.^16^ Data indicates that in 2022 43% of adults worldwide were overweight, while 16% (890 million) were obese, leading to an increased risk of hypertension, dyslipidemia, type 2 diabetes, coronary artery disease, stroke, certain cancers, and higher overall mortality.^17^

The ECS significantly influences appetite regulation, although its full impact remains unknown. Current evidence, based on studying the effect of introducing exogenous cannabinoids such as delta 9‐tetrahydrocannabinol (THC) or CBR1 blockers in humans and animals, suggests that the ECS plays a central role in energy balance, both centrally and peripherally.^18^, ^19^ Studies focusing on ECS regulation in humans have been scarce due to its complexity and ubiquitous nature, therefore more extensive research is required before its full potential is revealed.

### 3.1 Cannabinoid receptors 1

**FIGURE 2:**
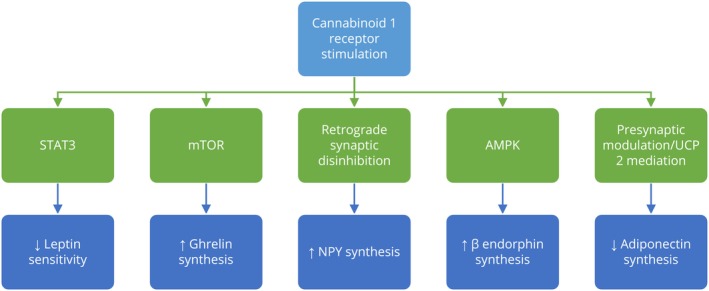
The Role of CB1 receptor stimulation in appetite regulation and obesity. CB1 receptor stimulation promotes orexigenic pathways leading to the increase in the synthesis of molecules such as ghrelin, NPY, and β‐endorphin, which are associated with increased appetite and potential weight gain. Conversely, CB1 receptor activation decreases anorexigenic signals by reducing leptin sensitivity and adiponectin synthesis, diminishing the body's ability to suppress appetite and regulate energy balance. AMPK ‐ AMP‐activated protein kinase; mTOR ‐ mammalian target of rapamycin; NPY ‐ neuropeptide Y; STAT3 ‐ signal transducer and activator of transcription 3; UCP2 ‐ mitochondrial uncoupling protein 2.

Endocannabinoids are crucial regulators of energy homeostasis, promoting energy storage by increasing appetite and food intake through central mechanisms, while also enhancing lipogenesis and glucose uptake in peripheral tissues, mainly via CBR1.^12^ In animal models, antagonism of CBR1 has been shown to decrease food intake.^20^ Furthermore, chronic CBR1 blockade not only affects food intake and body weight but also improves insulin and leptin sensitivity, enhances glucose and lipid profiles, and reduces hepatic steatosis and fibrosis in rodent models.^21^, ^22^, ^23^, ^24^ It is suggested this effects are modulated by different mediators (Figure 2).

Leptin, an anorexigenic hormone produced by adipocytes, plays a crucial role in appetite regulation, partly by interacting with the ECS. It decreases endocannabinoid synthesis by reducing calcium influx and downregulates CBR1 expression in the hypothalamus, leading to a decreased appetite.^12^, ^19^, ^25^, ^26^, ^27^, ^28^ Additionally, a lack of CBR1 stimulation enhances leptin sensitivity, whereas CBR1 stimulation reduces leptin's effect in the hypothalamus by inhibiting STAT3, a key intracellular effector of leptin.^25^, ^29^ However, in the context of obesity, leptin resistance disrupts its inhibitory effect on the ECS, resulting in increased food intake.^12^ In animal models, peripheral CBR1 inverse agonists have been found to reverse leptin resistance and reduce plasma leptin levels by decreasing its production in adipocytes, potentially through direct effects on CBR1 in adipose tissue or by enhancing sympathetic tone and leptin clearance.^30^

Food deprivation, contrastingly, triggers the release of endocannabinoids in the hypothalamus and limbic forebrain, activating CBR1 and stimulating appetite, in part through interactions with other orexigenic and anorexigenic signals.^5^, ^31^, ^32^ Activation of CBR1 stimulates the production of the orexigenic neuropeptide Y (NPY) in NPY/AgRP neurons within the arcuate nucleus, possibly by retrograde synaptic disinhibition.^33^, ^34^, ^35^ Interestingly, while CBR1 activation has been shown to increase activity in anorexigenic pro‐opiomelanocortin (POMC) neurons, subsequent research revealed that it primarily enhances the production of β‐endorphin, a supposed orexigenic peptide, without an increase in the anorexigenic α‐melanocyte‐stimulating hormone (α‐MSH).^36^ Additionally, the orexigenic effects of ghrelin, another appetite‐stimulating hormone, produced in the gastrointestinal system, depend on a functional ECS.^37^ Ghrelin increases the production of 2‐AG by raising intracellular Ca^2+^ levels, while CBR1 activity modulates the production of ghrelin via mTOR pathway.^27^, ^38^, ^39^

Peripherally, the ECS regulates food intake through the vagus nerve and modulates nutrient processing in the gastrointestinal (GI) tract.^14^, ^40^, ^41^, ^42^, ^43^ It was shown that the stimulation of CBR1 in the GI tract influences gastric secretion, gastric emptying, and intestinal motility.^40^ On the other hand, activation of the CBR1 receptors in fat cells promotes lipogenesis and inhibits the production of adiponectin, a cytokine secreted by adipocytes involved in regulating glucose levels, insulin sensitivity, and lipid metabolism. Low levels of adiponectin, possibly caused by a CB1‐mediated decrease in AMPK activity leading to a decrease in mRNA expression, are associated with several metabolic conditions, including obesity.^44^, ^45^, ^46^, ^47^

### 3.2 Cannabinoid receptors 2

CBR2 agonism in animal models has been associated with reduced food intake, although the findings are not entirely consistent.^27^ A key feature of CBR2 receptors is their involvement with dopamine neurons in the ventral tegmental area, where they influence the reward pathway, particularly in response to hedonic food intake.^7^ Studies in animals suggest that reduced CBR2 signaling (receptor ablation or antagonist administration) leads to increased food intake, greater adipocyte mass, and obesity, while enhanced CBR2 stimulation (overexpression or agonist administration) results in decreased food intake, reduced body weight, and improved insulin sensitivity.^7^, ^48^, ^49^, ^50^, ^51^ CBR2 agonism shows potential for reducing obesity‐associated inflammation; however, some studies report conflicting results, highlighting the need for further research.^52^, ^53^

### 3.3 Peroxisome proliferator‐activated receptors

Endocannabinoids also activate peroxisome proliferator‐activated receptors (PPARs), a family of nuclear receptors crucial for regulating gene expression, particularly the α and γ isoforms, which play significant roles in lipid metabolism.^54^, ^55^ It is proposed that PPAR activation partly mediates the neuroprotective, analgesic, anti‐inflammatory, metabolic, anti‐tumor, and cardiovascular effects of cannabinoids. These effects are achieved through complex interactions, with some compounds already being investigated that act as dual CBR1 antagonists/PPARα agonists or CBR2/PPARγ agonists.^56^, ^57^

### 3.4 ECS dysregulation

Current evidence links human obesity to the ECS dysregulation. ECS activation, increased endocannabinoid levels (both centrally and peripherally), and CBR1 up‐regulation, may be driven by high‐fat diets that increase the availability of polyunsaturated fatty acids, precursors for endocannabinoid biosynthesis.^14^, ^58^, ^59^, ^60^, ^61^, ^62^, ^63^, ^64^ The ingestion of highly palatable food activates brain reward circuits with the release of dopamine, endocannabinoids, and opiates, which induces a persistent stimulation of hypothalamic hunger signals and inhibition of satiety mediators. Therefore, obesity may be aggravated by the effect of the endocannabinoid activation in the nucleus accumbens, hippocampus, and entopeduncular nucleus, which are suspected to be directly involved in reward pathways related to hedonic eating.^14^, ^65^, ^66^ Genetic factors, such as the FAAH 385 A/A missense polymorphism, which reduces FAAH enzymatic activity, may also contribute to ECS dysregulation in obesity.^14^, ^26^, ^67^

An overview of the studies on the ECS and its impact on food intake, energy metabolism, and obesity is presented in Table 1.

**TABLE 1:** An overview of the studies on the endocannabinoid system and its impact on food intake, energy metabolism, and obesity.

Study	Main results	Study model
Kirkham TC (2005)	Reviewed the role of endocannabinoids in regulating appetite and body weight, highlighting the behavioral pharmacology aspects and how endocannabinoids influence feeding behavior and energy homeostasis.	Review (Animal and Human Studies)
Di Marzo V, Matias I (2005)	Discussed the endocannabinoid control of food intake and energy balance, emphasizing the complex interactions within the endocannabinoid system that regulate metabolism and appetite.	Review (Animal and Human Studies)
Viveros M, De Fonseca F, Bermudez‐Silva F, McPartland J (2008)	Discusses the crucial role of the ECS in regulating food intake and energy metabolism, highlighting its evolutionary, developmental, and pathological aspects. It connects to obesity by explaining how the ECS, particularly through the activation of CB1 receptors, influences hunger and metabolism, contributing to weight gain and metabolic disorders, thereby presenting a target for obesity treatment interventions.	Review (Animal and Human Studies)
Harrold JA, Williams G (2003)	Discussed the role of the cannabinoid system in both homeostatic and hedonic control of eating, indicating its influence on feeding behavior through central mechanisms.	Review (Animal and Human Studies)
Di Marzo V (2008)	Examined the involvement of the endocannabinoid system in obesity and type 2 diabetes, suggesting that dysregulation of this system contributes to these metabolic disorders.	Review (Animal and Human Studies)
Cota D, Marsicano G, Tschöp M, et al. (2003)	Found that the endogenous cannabinoid system affects energy balance via central orexigenic drive and peripheral lipogenesis, highlighting its dual role in energy homeostasis.	Animal Studies
Szanda G, Jourdan T, Wisniewski É, et al. (2023)	Demonstrated that CB1R inhibits hypothalamic leptin signaling via β‐arrestin1 in complex with TC‐PTP and STAT3, indicating a mechanism by which cannabinoids influence appetite regulation.	Cell and Animal Studies
Sipe JC, Waalen J, Gerber A, Beutler E (2005)	Identified an association between overweight and obesity with a missense polymorphism in FAAH 385 A/A, implicating genetic factors in the endocannabinoid system's influence on body weight which may provide indirect evidence to support cannabinoid antagonist treatment strategies in overweight disorders.	Human Studies
Miralpeix C, Reguera AC, Fosch A, et al. (2021)	Discussed the role of hypothalamic endocannabinoids in obesity and the challenges in understanding their precise mechanisms in the context of energy balance and metabolic regulation.	Review (Animal and Human Studies)
Watkins BA, Kim J (2015)	Reviewed the endocannabinoid system's role in directing eating behavior and macronutrient metabolism, emphasizing its influence on dietary habits and nutrient processing. The main finding is that PUFAs can modulate the ECS, which in turn affects energy balance, appetite, and overall metabolic health. This modulation by PUFAs has implications for managing obesity and metabolic disorders, particularly as individuals age, due to the ECS's role in regulating energy homeostasis and nutrient processing.	Review (Animal and Human Studies)
Owyang C, Heldsinger A (2011)	Examines the mechanisms through which the vagus nerve regulates feeding behavior. It highlights the roles of cholecystokinin (CCK) and leptin in signaling satiety. CCK primarily controls short‐term food intake, while leptin influences long‐term eating behavior and body weight. The study found that the endocannabinoid system interacts with these mechanisms, particularly involving the vagus nerve, to modulate appetite and energy balance.	Review (Animal and Human Studies)
Kowalski CW, Ragozzino FJ, Lindberg JEM, et al. (2020)	Found that cannabidiol activation of vagal afferent neurons requires TRPA1, suggesting a novel pathway for the endocannabinoid system in modulating satiety. The study suggests that CBD's activation of vagal afferent neurons could influence appetite regulation and energy balance through its effects on the endocannabinoid system. By modulating these pathways, CBD may play a role in managing obesity and related metabolic disorders.	Animal Studies
DiPatrizio NV, Astarita G, Schwartz G, et al. (2011)	The study found that the endocannabinoid system in the gut regulates dietary fat intake by mobilizing endocannabinoids (2‐AG and AEA) in response to fat exposure. This process involves the vagus nerve and CBR1 receptors in the gut. Targeting the gut endocannabinoid system could help reduce overeating of fatty foods and address obesity.	Animal Studies
Jean‐Pierre D, Alain G, Lars S (2005)	Investigated the effects of Rimonabant on metabolic risk factors in overweight patients with dyslipidemia, showing significant impacts on weight and lipid profiles.	Human Studies
Di Marzo V, Goparaju SK, Wang L, et al. (2001)	Demonstrated that leptin‐regulated endocannabinoids are involved in maintaining food intake, revealing an interaction between leptin signaling and endocannabinoid activity.	Animal Studies
Sipe JC, Scott TM, Murray S, et al. (2010)	The study found that carriers of the FAAH 385 A mutant alleles have significantly elevated plasma levels of AEA and related N‐acylethanolamines (NAEs) compared to wild‐type carriers, indicating endocannabinoid system activation. This elevation was particularly notable in severely obese individuals, suggesting a link between the FAAH 385 A allele and severe obesity. These findings highlight the potential of targeting the endocannabinoid system for novel obesity treatments.	Human Studies
Richey JM, Woolcott O (2017)	Reviewed the therapeutic potential of targeting the endocannabinoid system in obesity and associated diseases, highlighting its regulatory role in energy balance.	Review (Animal and Human Studies)
Matias I, Gatta‐Cherifi B, Tabarin A, et al. (2012)	Measured endocannabinoids in human saliva as potential biomarkers of obesity, proposing a non‐invasive method for assessing endocannabinoid activity.	Human Studies
Blüher M, Engeli S, Klöting N, et al. (2006)	The study found that in individuals with abdominal obesity, the endocannabinoid system is dysregulated in peripheral and adipose tissues. This dysregulation is characterized by altered levels of endocannabinoids, which are implicated in the development and maintenance of obesity.	Human Studies
Osei‐Hyiaman D, DePetrillo M, Pacher P, et al. (2005)	Found that endocannabinoid activation at hepatic CB1 receptors stimulates fatty acid synthesis and contributes to diet‐induced obesity, highlighting a liver‐specific pathway.	Animal Studies
Kim J, Li Y, Watkins BA (2013)	Reviewed the relationship between dietary PUFA, endocannabinoids, and obesity, suggesting dietary interventions targeting the endocannabinoid system. Specifically, PUFAs can modulate endocannabinoid levels, potentially offering a dietary approach to manage obesity.	Review (Animal and Human Studies)
Hansen HS, Artmann A (2008)	Explored the interplay between endocannabinoids and nutrition, emphasizing their role in metabolic regulation and dietary effects. The study highlights that certain dietary fats can modulate the levels of endocannabinoids, which play a crucial role in energy balance and metabolism, suggesting that dietary interventions targeting the ECS could be a potential strategy for managing obesity.	Review (Animal and Human Studies)
Monteleone P, Piscitelli F, Scognamiglio P, et al. (2012)	Found that hedonic eating is associated with increased peripheral levels of ghrelin and 2‐AG, linking pleasure‐driven eating to endocannabinoid activity, which could have implications for understanding obesity.	Human Studies
Aguilera Vasquez N, Nielsen DE (2022)	Suggests that the ECS plays a significant role in regulating eating behaviors, particularly in the context of obesity. The study reviews current evidence indicating that dysregulation of the ECS can influence appetite, food intake, and energy balance, thereby contributing to the development and maintenance of obesity.	Review (Animal and Human Studies)
Engeli S, Böhnke J, Feldpausch M, et al. (2005)	Discussed that peripheral ECS is significantly activated in individuals with obesity. This activation is characterized by elevated levels of endocannabinoids in the peripheral tissues, which are associated with various metabolic alterations contributing to obesity.	Human Studies

## 4 THERAPEUTIC APPROACHES AND CHALLENGES

The widespread distribution of the ECS throughout the human body, its on‐demand synthesis, and specific activation during pathological states, make selective pharmacological intervention within the ECS highly challenging. The success of CBR1 blocker, rimonabant, in treating obesity was demonstrated in clinical trials.^44^, ^68^, ^69^ However, the systemic use of cannabinoid receptor modulators, which indiscriminately affect the function of all cannabinoid receptors across different tissues can disrupt the normal function of the ECS in non‐target cells. This disruption poses a significant risk of developing adverse effects, such as the intoxication associated with CBR1 agonists and the psychological side effects caused by CBR1 antagonists or inverse agonists.^62^, ^70^ Selective pharmacological targeting that ensures the anti‐obesity effect while minimizing the risk of adverse effects is therefore essential in developing anti‐obesity drugs aimed at the ECS.

### 4.1 Rimonabant

The discovery of the cannabinoid receptors opened the door to pharmacological intervention within the ECS. Rimonabant, an inverse agonist of the CBR1, was shown to suppress the endogenous activation of the ECS both centrally and peripherally.^44^, ^68^, ^71^, ^72^ In clinical trials, rimonabant has shown promise as an anti‐obesity agent achieving weight reduction and improvements in lipid and HbA1C levels.^44^, ^68^, ^71^ It is estimated that half of the effect of rimonabant on HDL‐cholesterol and triglycerides levels was independent of weight loss, possibly due to stimulation of adiponectin production through CBR1 inhibition.^44^, ^68^, ^69^, ^71^ After approval as a weight‐loss drug in Europe in 2006, in 2008, before receiving FDA approval in the USA, it was withdrawn from the European market due to severe psychiatric adverse effects, including depression, anxiety, and even suicidal ideation.^62^, ^70^, ^72^, ^73^ Consequently, other clinical trials of drugs with a similiar mechanism of action were terminated.^62^, ^70^

### 4.2 Drugs in development

After rimonabant was pulled from the market due to psychological adverse effects, the search for a more selective agent began. One approach is to develop peripherally restricted CB1 antagonists, which minimize CNS side effects,^67^, ^70^, ^74^ and CB1 negative allosteric modulators (NAMs),^75^ which produce more specific receptor modulation with a better safety profile. Additionally, the potential role of CB2 receptors in energy homeostasis and inflammation is being investigated since CBR2 are expressed in the brain regions involved in appetite regulation, but are not involved in mood regulation.^49^ CBR2 also influence fat tissue cells, mediating the adipocyte‐induced inflammatory response,^76^ as well as browning of white adipose tissue, a process typically inhibited in obesity.^77^ Besides modulating ECS activity by targeting CBR, another approach is via the enzymes responsible for the synthesis or degradation of the endocannabinoids, namely FAAH, MAGL, and DAGL. However, due to complex and tissue‐specific activity of ECS enzymes, predicting the outcomes of such interventions is challenging.^4^, ^6^, ^27^, ^78^

New therapeutic strategies are currently being explored with several drugs currently in the pipeline. The first biologic drug targeting the ECS, namacizumab, a negative allosteric modulating antibody (NAMA) that stabilizes CBR1 in an inactive conformation, was approved to initiate a phase 1 of the clinical trial in the treatment of non‐alcoholic steatohepatitis (NASH) and diabetic nephropathy.^74^ Moreover, INV‐101, a peripheral CBR1 inverse agonist, has been approved to start phase 1 of the clinical trial for the treatment of Prader‐Willi syndrome characterized by overactivated ECS, which leads to excessive weight gain, hyperphagia, and neurodevelopmental symptoms.^79^ A Phase 2 trial is currently evaluating the efficacy of once‐daily oral cannabis for weight loss in obese patients, with results expected in 2026.^80^ There are some data suggesting that cannabis may influence weight loss, potentially through its effects on the CBR2,^81^ although the evidence is not yet conclusive.^82^, ^83^

### 4.3 Advantages of targeting ECS in obesity

Taking into account all of the above, targeting the ECS for anti‐obesity treatments offers a unique therapeutic approach that differs from existing drugs, potentially addressing obesity through multiple mechanisms while minimizing some limitations of current therapies. As described, the ECS is deeply involved in the regulation of food intake, energy storage, and glucose and lipid metabolism. Drugs targeting the ECS could modulate these pathways more effectively than current anti‐obesity drugs, which often focus on singular aspects such as appetite suppression or fat absorption. In addition, targeting the ECS might help restore leptin sensitivity, thereby improving the body's natural ability to regulate weight. Moreover, CBR2 activation shows promise in reducing obesity related inflammation potentially providing metabolic benefits beyond weight loss.

## 5 CONCLUSION

Despite these promising observations, the main fault of any attempted endocannabinoid manipulation lies in the ubiquitous nature of the ECS across the human body, and its involvement in various physiological processes, which in turn carries a high risk of unwanted adverse effects. Future research endeavors may warrant selective ECS manipulation in specific tissues, in an attempt to circumnavigate this issue.

## 6 NOMENCLATURE STATEMENT

Key protein targets and ligands in this article are hyperlinked to corresponding entries in http://www.guidetopharmacology.org, the common portal for data from the IUPHAR/BPS Guide to PHARMACOLOGY (Harding et al., 2018), and are permanently archived in the Concise Guide to PHARMACOLOGY 2019/20 (Alexander et al., 2019).^84^, ^85^

## AUTHOR CONTRIBUTIONS

All authors contributed substantially to the conception of the work. MK and IR wrote the first draft of the work. RL performed critical revision. All authors gave final approval of the version to be published.

## CONFLICT OF INTEREST STATEMENT

None to declare.

7

## ETHICS STATEMENT

None.

## Data Availability

Data are available upon reasonable request from the authors.
